# Development and Validation of a Clinical Prognostic Nomogram for Esophageal Adenocarcinoma Patients

**DOI:** 10.3389/fonc.2021.736573

**Published:** 2021-09-02

**Authors:** Chen-ye Shao, Yue Yu, Qi-fan Li, Xiao-long Liu, Hai-zhu Song, Yi Shen, Jun Yi

**Affiliations:** ^1^Department of Cardiothoracic Surgery, Nanjing Hospital of Chinese Medicine, Nanjing, China; ^2^Department of Thoracic Surgery, The First Affiliated Hospital of Nanjing Medical University, Nanjing, China; ^3^Department of Thoracic Surgery, The First Affiliated Hospital of Soochow University, Suzhou, China; ^4^Department of Cardiothoracic Surgery, Jinling Hospital, Medical School of Nanjing University, Nanjing, China; ^5^Department of Medical Oncology, Jinling Hospital, Medical School of Nanjing University, Nanjing, China

**Keywords:** esophagus diseases, esophageal adenocarcioma (EAC), cancer staging, thoracic oncology, esophagus, clinical staging system

## Abstract

**Background:**

Clinical staging is essential for clinical decisions but remains imprecise. We purposed to construct a novel survival prediction model for improving clinical staging system (cTNM) for patients with esophageal adenocarcioma (EAC).

**Methods:**

A total of 4180 patients diagnosed with EAC were extracted from the Surveillance, Epidemiology, and End Results (SEER) database and included as the training cohort. Significant prognostic variables were identified for nomogram model development using multivariable Cox regression. The model was validated internally by bootstrap resampling, and then subjected to external validation with a separate cohort of 886 patients from 2 institutions in China. The prognostic performance was measured by concordance index (C-index), Akaike information criterion (AIC) and calibration plots. Different risk groups were stratified by the nomogram scores.

**Results:**

A total of six variables were determined related with survival and entered into the nomogram construction. The calibration curves showed satisfied agreement between nomogram-predicted survival and actual observed survival for 1-, 3-, and 5-year overall survival. By calculating the AIC and C-index values, our nomogram presented superior discriminative and risk-stratifying ability than current TNM staging system. Significant distinctions in survival curves were observed between different risk subgroups stratified by nomogram scores.

**Conclusion:**

The established and validated nomogram presented better risk-stratifying ability than current clinical staging system, and could provide a convenient and reliable tool for individual survival prediction and treatment strategy making.

## Introduction

Esophageal cancer (EC) account for over 500,000 cancer deaths annually, which constitute a major global health problem (1). According to the Global Cancer Statistics of 2020, EC has the 8th highest incident and is the 6th leading cause of cancer-related deaths worldwide (2). Esophageal Squamous Cell Carcinoma (ESCC) and Esophageal Adenocarcioma (EAC) are two common histologic subtypes of EC (3). New to the eighth edition of the American Joint Committee on Cancer (AJCC) Cancer Staging for cancer of the esophagus and esophagogastric junction are independent, temporally related cancer classifications: clinical (cTNM), pathologic (pTNM), and postneoadjuvant pathologic (ypTNM) stage groups (4–7). Moreover, classifications for the two major histopathologic cell types are no longer shared. Except for the ypTNM classifications, esophageal squamous cell carcinoma and esophageal adenocarcioma present independent staging groups in clinical (cTNM) and pathological (pTNM) classifications (7).

The eighth edition of the AJCC Cancer clinical staging system for EAC is based on the extent of primary tumor (clinical T status, cT), involved reginal lymph nodes (clinical N status, cN) and distant metastasis (clinical M status, cM) (4). However, substantial heterogeneity exists among patients in each subgroup (8–10). To obtain a novel clinical staging system with better discriminative ability in prognostic prediction, we previously constructed a nomogram model for ESCC patients from the SEER (Surveillance, Epidemiology, and End Results) database based on multiple clinical characteristics (11). By adding potential prognostic factors which are not included in current TNM system (sex, age, tumor size and grade), superior risk-stratifying ability was observed in the established nomogram. Similarly, the present study was designed to develop a prognostic nomogram for patients with EAC based on SEER database and to evaluate its reliability and feasibility through independent external cohorts.

## Methods

The SEER database (www.seer.cancer.gov) is an authoritative population-based cancer registry that covers approximately 28% of the US population. A total of 29623 patients with EAC (from 1988 to 2017) were identified from SEER database, of which 4180 cases met our inclusion criteria and were included as the training cohort. The following variables were extracted: sex, age, tumor location, tumor size, grade, cT status, cN status, cM status, cTNM stage (based on the AJCC 8th staging system) and follow-up data. To examine the reliability and feasibility of the prognostic nomogram model, an external validation cohort was composed of 886 patients from Jinling Hospital of Nanjing Medical University (n=695, from January 2008 to May 2018) and the First Affiliated Hospital of Nanjing Medical University (n=191, from November 2010 to September 2017). Patients were included based on the following criteria: (1) confirmed pathology of primary esophageal adenocarcinoma; (2) patients diagnosed with EAC after 2004 (to obtain more accurate clinicopathological information). Patients were excluded based on the following criteria: (1) a history of definitive chemoradiotherapy (CRT) or radiation therapy (RT); (2) incomplete information or postoperative mortality. The inclusion and exclusion criteria of the training cohort and external validation cohort were the same. OS was defined as the time interval between the first clinical decision and death. The ethical approval was approved by the Research Ethics Committee.

### Determination of Clinical Stage

Clinical T status was determined by endoscopic ultrasound (EUS) and chest and abdominal computed tomography; clinical N (non-peritumoral) status was determined by the combination of the EUS-FNA and computed tomography-positron emission tomography (PET-CT); clinical M status was determined by PET-CT. Furthermore, tumor location, histopathologic cell types and grade were determined by esophagogastroscopy and biopsy. When differences were observed among radiographic outcomes, we recorded the farthest reported tumor extension as cT. Similarly, the cN was defined as the farthest involved reginal lymph nodes. When differences were observed between imaging outcomes and biopsy pathological outcomes, the outcomes of pathological biopsy of lymph nodes were adopted first. These criteria were in accordance with the SEER database.

### Development and Validation of the Nomogram

The training cohort (n=4180) was utilized for developing the nomogram. The survival rate was calculated using the Kaplan-Meier method, and the differences between curves were assessed by the log-rank test. Univariate Cox regressions were constructed to identify prognostic variables. Variables with a p value <0.05 in univariate survival analysis were further analyzed in multivariate analysis. Multivariate analysis was performed by the Cox proportional hazard model using the forward procedure based on likelihood ratio for variable selection. Then, a nomogram was developed on the basis of the consequences of the multivariable analysis. Each subgroup of candidate variables was assigned certain prognostic points. The instructions for using the nomogram: By drawing a line upward, the individual points could be located on the point scale (ranging from 0-100). By summing up these points of each variable, the 1-, 3-, 5-year overall survival rates were predicted by drawing a straight line from total point scale to the corresponding survival scale. For the internal validation, 1000 bootstrap resamples in the training cohort were performed. Moreover, the established nomogram was validated using an independent external validation cohort (n=886). The discrimination ability of each prognostic system was assessed using the C-index. By evaluating the consistency between predicted survival probability and actual survival proportion, calibration curves were plotted using the average K-M estimates to assess the performance of the predictive model.

### Novel Risk Grouping Method and Survival Analysis

By dividing the forementioned prognostic points by 10 (for prognostic points estimation, please see the instructions for nomogram), we obtained the prognostic scores of each subgroup and individual total prognostic score based on the nomogram. Classification and regression tree (CART) model was then developed to construct a decision tree model using the total prognostic score of patients. The root node of CART model contained all cases in the training cohort and was separated into 2 subgroups basing on the Gini impurity index. By utilizing the recursive iterative algorithm, these subgroups would keep splitting until the survival of patients in the subgroup was homogeneous. According to the CART model, the cutoff values of total prognostic score were obtained. These cutoff values were then applied to different TNM categories to group patients into different risk subgroups. Kaplan-Meier survival analysis was performed to assess the significance of the survival difference between different subgroups for assessing the discrimination capability of the novel risk grouping method.

### Statistical Analysis

Statistical analysis to identify prognostic variables and develop CART model was performed using the SPSS 22.0 software package (SPSS, inc., Chicago, IL). The R packages ‘survival’, ‘foreign’, and ‘rms’ were used for nomogram construction and evaluation in R 3.3.2 (http://www.r-project.org). For all statistical testing, we used a 2-sided significance level (alpha) of 0.05.

## Results

### Patient Characteristics

A total of 29623 patients with EAC were extracted from the SEER database. For these 29623 cases, patients who accepted definitive CRT or RT (n=1667), with incomplete information (n=22897) and with postoperative mortality (n=879) were excluded. Thus, 4180 patients met the inclusion criterion and were eventually included as the training cohort. Basing on the AJCC 8th staging system, patients were grouped as follows: c I: 569 (13.6%); c IIA: 114 (2.7%); c IIB: 238 (5.7%); c III: 1618 (38.7%); c IVA: 470 (11.2%); c IVB: 1171 (28.0%). Of the 1317 cases in the validation cohort, patients who accepted definitive CRT or RT (n=101), with incomplete information before treatment (n=315) and with postoperative mortality (n=15) were excluded. Finally, the validation cohort comprised 886 patients with EAC diagnosed between 2008 and 2018, which included 695 patients from Jinling Hospital of Nanjing Medical University, and 191 patients from the First Affiliated Hospital of Nanjing Medical University. Patients were grouped as follows according to the AJCC 8th staging system: c I: 120 (13.5%); c IIA: 30 (3.4%); c IIB: 44 (5.0%); c III: 337 (38.0%); c IVA: 98 (11.1%); c IVB: 257 (29.0%). The details of patient characteristics are listed in **Table 1**.

**Table 1:** Demographic characteristics of the training and validation cohorts.

Characteristics	Training cohort (n=4180)	Validation cohort (n=886)	P value
Sex			
Male	3630	775	0.61
Female	550	111	
Age			
<50	272	49	0.46
50-60	751	148	
60-70	1335	279	
70-80	1095	238	
≥80	727	172	
Tumor location			
Upper	45	7	0.74
Middle	290	62	
Lower	3845	817	
Tumor size (cm)			
< 2cm	588	124	0.55
2-5cm	2116	443	
5-8cm	1023	213	
≥8cm	453	106	
Clinical T status			
T1	948	221	0.43
T2	574	116	
T3	1771	344	
T4	887	205	
Clinical N status			
N0	1711	356	0.38
N1	1756	391	
N2	519	108	
N3	194	31	
Clinical M status			
M0	3009	629	0.55
M1	1171	257	
Clinical TNM stage			
I	569	120	0.82
IIA	114	30	
IIB	238	44	
III	1618	337	
IVA	470	98	
IVB	1171	257	
Grade			
G1+G2	1966	417	0.98
G3	2214	469	

G1, Well; G2, Moderate; G3, Poor/Undifferentiated.

There were 3375 deaths over an average follow-up duration of 38.7 months (range, 1.1-118.8 months) in the training cohort. 734 deaths were observed over an average follow-up duration of 30.9 months (range, 1.1-99.2 months) in the validation cohort. The training cohort had median OS of 13 months, with 1-, 3-, and 5-year OS rates of 51.37%, 24.02% and 12.47%, respectively. The validation cohort had a median OS of 13 months, with 1-, 3-, and 5-year OS rates of 51.13%, 23.36% and 14.06%, respectively.

### Clinical Nomogram for OS

**Figure 1:**
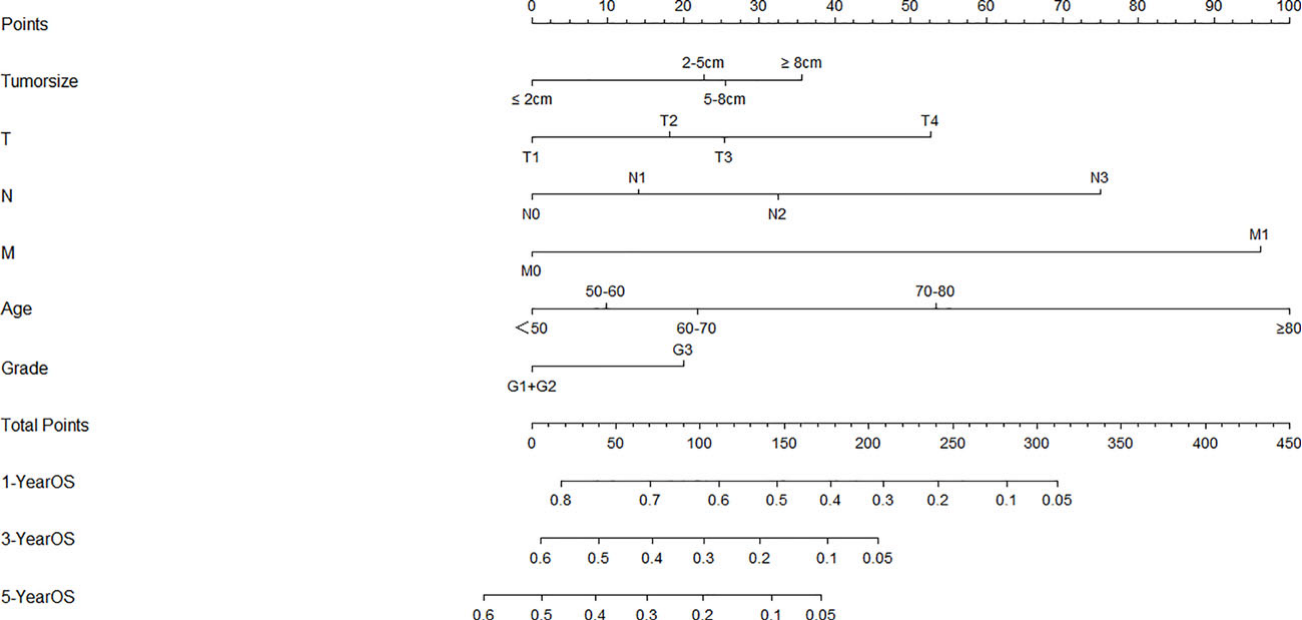
Nomogram for overall survival developed from the training cohort. T, clinical T status; N, clinical N status; M, clinical M status.

The univariate analysis indicated that six variables (including age, tumor size, cT status, cN status, cM status and grade, P < 0.05) were significantly associated with OS (**Table 2**). These six independent risk factors were selected for the multivariable analysis using a Cox regression. The multivariate logistic analysis confirmed that age≥60, tumor size≥2cm, advanced T, N and M categories and poor/undifferentiated tumor grade were independent prognostic variables. A prognostic nomogram integrating aforementioned significant variables was constructed (**Figure 1**). By dividing the forementioned prognostic points by 10 (for prognostic points estimation, please see **Figure 1**), we obtained the prognostic scores of each subgroup (ranging from 0-10, **Table 3**).

**Table 2:** Results of univariable and multivariate Cox proportional hazards regression analysis for overall survival in the training cohort.

Characteristics	Univariable Analysis	Multivariable Analysis		
	P value	Hazard Ratio	95% CI	P value
Sex				
Male	0.60	Reference	0.96-1.18	0.23
Female		1.06		
Age				
<50	<0.001	Reference		
50-60		1.08	0.93-1.28	0.29
60-70		1.21	1.04-1.41	0.01
70-80		1.60	1.37-1.86	<0.001
≥80		2.40	2.05-2.81	<0.001
Tumor location				
Upper	0.18	Reference		
Middle		0.88	0.63-1.24	0.48
Lower		0.81	0.60-1.12	0.21
Tumor size (cm)				
<2cm	<0.001	Reference		
2-5cm		1.22	1.10-1.37	<0.001
5-8cm		1.25	1.11-1.41	<0.001
≥8cm		1.37	1.19-1.58	<0.001
Clinical T status				
T1	<0.001	Reference		
T2		1.18	1.04-1.33	0.009
T3		1.25	1.13-1.39	<0.001
T4		1.59	1.43-1.78	<0.001
Clinical N status				
N0	<0.001	Reference		
N1		1.14	1.05-1.23	0.002
N2		1.34	1.19-1.50	<0.001
N3		1.95	1.66-2.30	<0.001
Clinical M status				
M0	<0.001	Reference	2.16-2.55	<0.001
M1		2.35		
Grade				
G1+G2	<0.001	Reference	1.11-1.28	<0.001
G3		1.19		

G1, Well; G2, Moderate; G3, Poor/Undifferentiated; CI, confidence interval.

**Table 3:** The prognostic scores of each subgroup within variable.

Subgroups of each variable	Prognostic Score
Age	
<50	0
50-60	1.0
60-70	2.2
70-80	5.3
≥80	10
Tumor size (cm)	
≤2cm	0
2-5cm	2.3
5-8cm	2.6
≥8cm	3.6
Clinical T status	
T1	0
T2	1.8
T3	2.5
T4	5.3
Clinical N status	
N0	0
N1	1.4
N2	3.2
N3	7.5
Clinical M status	
M0	0
M1	9.6
Grade	
G1+G2	0
G3	2.0

### Performance of the Nomogram

**Figure 2:**
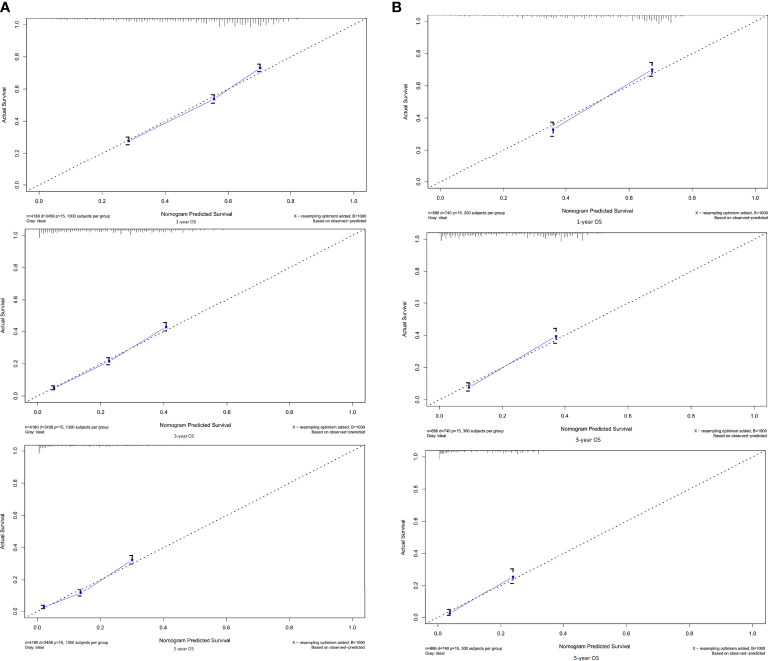
Calibration curves for 1-, 3-, 5-OS in the **(A)** training and **(B)** validation cohorts. By plotting nomogram-predicted overall survival on x-axis and actual observed overall survival on y-axis, the closer the drawn line is to 45 degrees, the better the calibration model is (it means the predicted probabilities are more identical to the actual outcomes).

In the training cohort, the C-index of the established nomogram to predict OS (0.68; 95% CI, 0.67-0.69) was significantly higher than that of the TNM staging system (0.63; 95% CI, 0.61-0.64; P<0.05). A similar result was observed in the external validation cohort. The C-index of the established nomogram (0.67; 95% CI, 0.66-0.69) was greater than that of the TNM staging system (0.61; 95% CI, 0.60-0.62; P<0.05). The calibration plots at 1-, 3-, and 5-year survival showed favorable consistency both in the training cohort (**Figure 2A**) and the validation cohort (**Figure 2B**) between the nomogram prediction and actual observation. AIC (Akaike information criterion) value was calculated for comparing the statistical fitness of different prognostic systems. The consequences (established nomogram *vs.* AJCC clinical staging system: 51661 *vs.* 51982) indicated that the established nomogram presented superior risk-stratifying capability.

### Novel Risk Grouping Method on the Basis of Individual Prognostic Score

**Figure 3:**
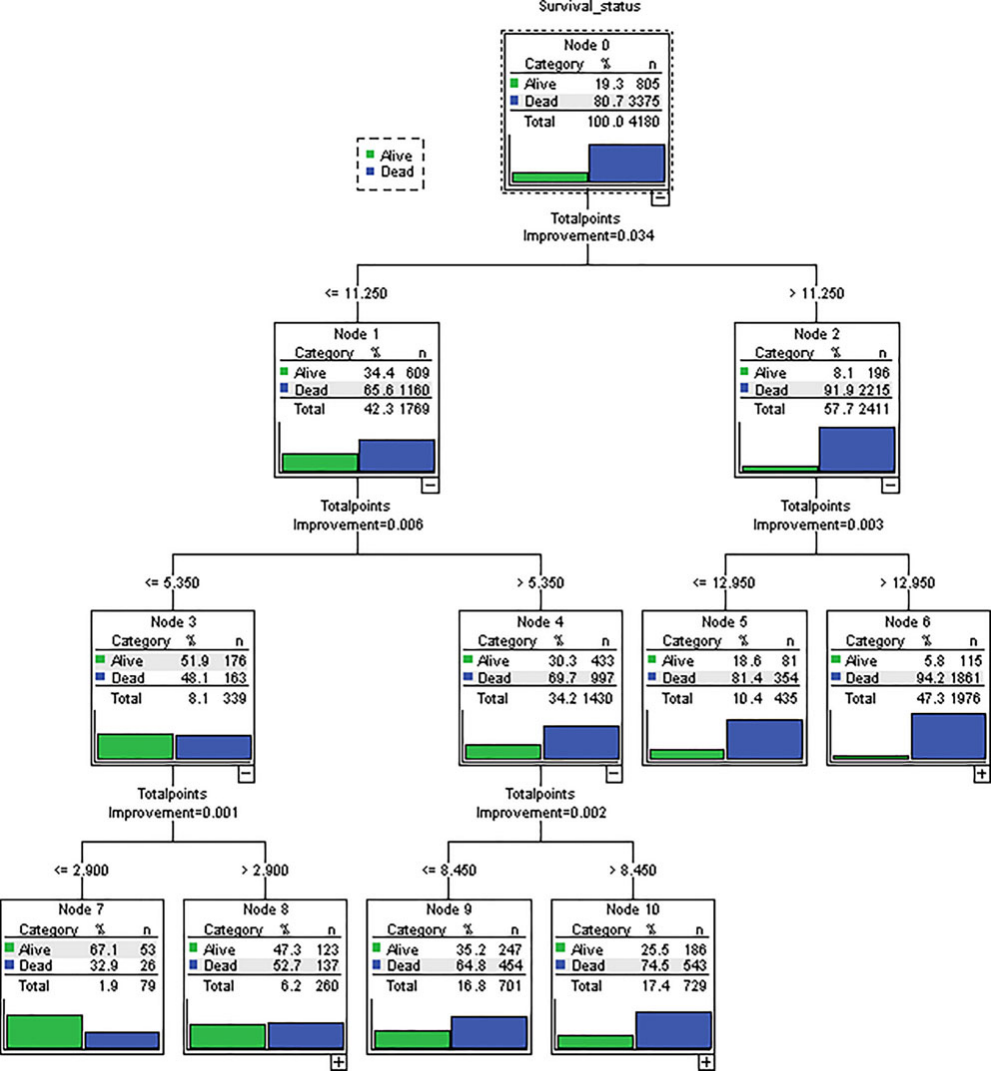
Novel risk grouping method based on individual prognostic sum-scores. Cutoff values were determined by constructing classification and regression tree (CART) models. We grouped patients into 6 risk groups. (0-2.90; 2.90-5.35; 5.35-8.45; 8.45-11.25; 11.25-12.95; > 12.95).

**Figure 4:**
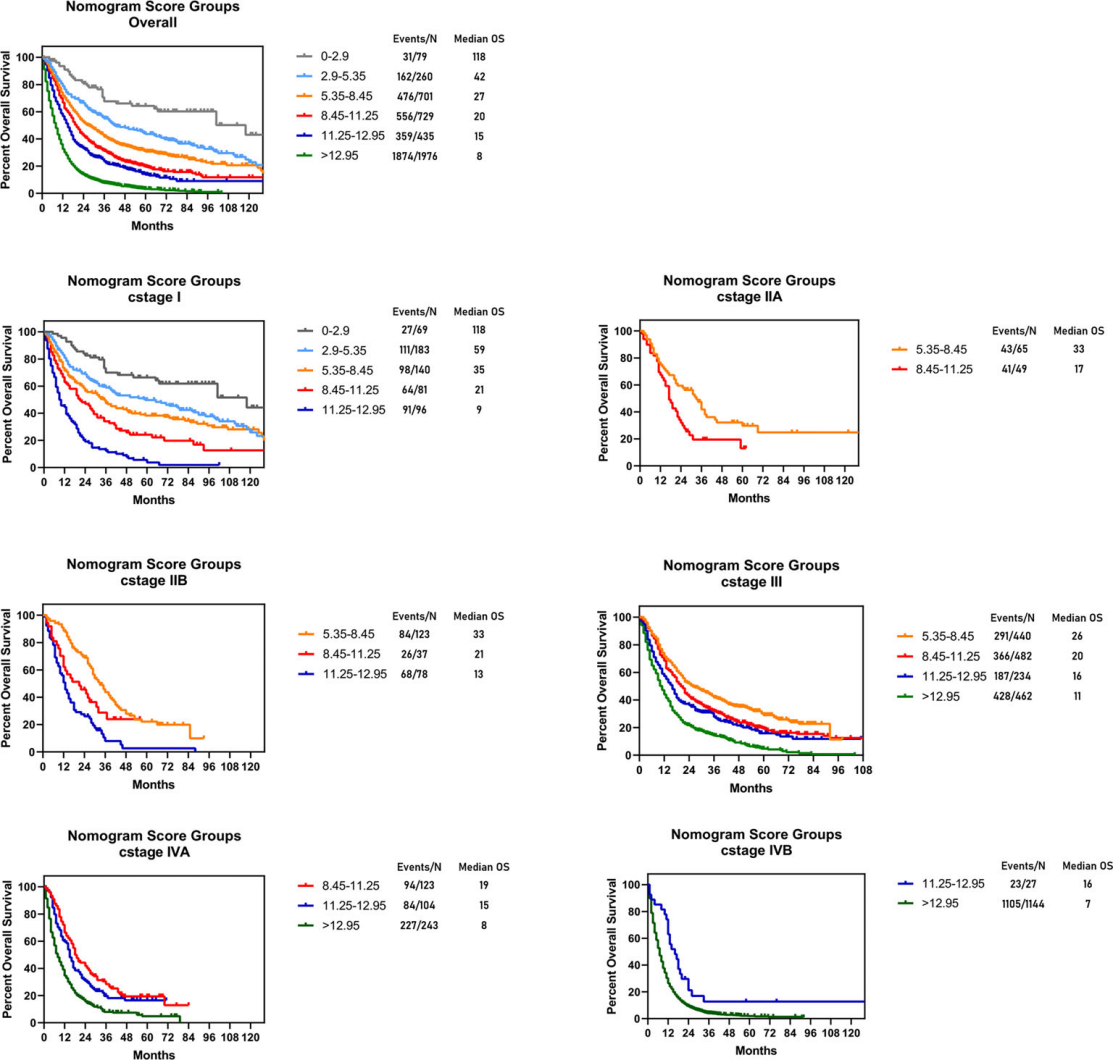
Risk group stratification within each TNM stage in the training cohort. Subgroups with fewer than 10 patients were omitted from the graphs.

The novel risk grouping method was developed based on the prognostic score by constructing classification and regression tree (CART) models (**Figure 3**). The recursive iterative algorithm was performed on the basis of the total prognostic scores for determining the cutoff values. We further divided the patients into six risk groups after sorting by total score (0-2.90; 2.90-5.35; 5.35-8.45; 8.45-11.25; 11.25-12.95; >12.95); each group presented a distinct prognosis. In the training cohort, the patients were predicted to have a significantly worse OS as the scores increased: 67.1%, 47.3%, 35.2%, 25.5%, 18.6% and 5.8%, respectively (p<0.05 between any two adjacent subgroups, **Table 4**). Survival curves were depicted among entire cohort and each AJCC clinical staging subcategory, respectively. As shown in **Figure 4**, the survival curves for OS revealed significant distinctions between any two subgroups in the overall and stage-stratified patients (according to the AJCC 8th clinical staging system) in the training cohort.

**Table 4:** Six groups with distinct prognosis were identified by classification and regression tree model.

Total prognostic score	5-Year Overall Survival (%)
0-2.90	67.1%
2.90-5.35	47.3%
5.35-8.45	35.2%
8.45-11.25	25.5%
11.25-12.95	18.6%
>12.95	5.8%

The cut-off values of total prognostic score were determined by the recursive iterative algorithm.

## Discussion

The eighth edition staging system separates classifications for the clinical (cTNM), pathologic (pTNM), and postneoadjuvant pathologic (ypTNM) stage groups (7). Current clinical staging for EAC based largely on physical examination, preoperative radiographic information, esophagoscopy and biopsy and is vital for treatment strategy making (12). However, EAC is remarkably heterogeneous in regard to survival of individual patients, prediction of survival using the TNM staging system is imprecise. To improve pathologic (pTNM) and postneoadjuvant pathologic staging system (ypTNM), several studies succeeded in providing better prognostic staging systems for EC patients by developing prognostic nomogram (13–17). To our knowledge, no predictive model is available for improving clinical staging system (cTNM) of EAC patients. Thus, in the present study, we constructed a preoperative nomogram for improving clinical staging. Moreover, based on easily-obtained variables, a novel risk grouping method was developed for individual survival prediction, which could help clinicians in treatment decision making. The nomogram showed satisfactory stratification capability and wide applicability when validated using external cohort and presented superior than the AJCC 8th staging system.

In this study, independent prognostic variables for EAC patients were revealed by the multivariate analysis, which included age, tumor size, cT status, cN status, cM status and grade. This result was in accordance with previous reports (14, 16, 18). The current AJCC 8th clinical staging system is based solely on cT status, cN status and cM status and does not incorporating other variables which could impact prognosis. We hypothesized that an improved prognostic grouping could be achieved by including additional parameters into our nomogram. To examine the performance of the established nomogram, an external validation cohort (n=886) was obtained from two institutions in China. The calibration curves showed satisfactory agreement between predicted survival probability and actual observation in the training cohort, which indicated good repeatability and reliability of the nomogram. Similar outcomes were also observed in the external Chinese validation cohort, which guaranteed the wide applicability of this nomogram despite ethnic and geographical differences. The C-index indicated that the established nomogram presented significant higher discriminate ability compared with the AJCC TNM staging system in the training cohort (C-index, 0.68 *vs* 0.63; P<0.05). Similar superiority was also found in the validation cohort (C-index, 0.67 *vs* 0.61; P<0.05). By calculating the AIC value, the prognostic model was revealed to yield a better prognostic stratification than AJCC TNM staging system (51661 *vs.* 51982).

By constructing classification and regression tree models, patients were stratified into 6 subgroups (0-2.90; 2.90-5.35; 5.35-8.45; 8.45-11.25; 11.25-12.95; >12.95) on the basis of individual sum-scores. Significant distinctions were observed in the survival curves in the entire cohort, and even among subcategories of the eighth edition of AJCC staging clinical system. It suggested that the current c I, c II, c III, c IVA and c IVB were heterogeneous groups and could be further stratified. The satisfied risk-stratifying ability indicated that our nomogram could divide patients into more homogeneous subgroups and had a potential complementary role to the current clinical staging system. This novel risk grouping method could help clinicians predict the individualized survival, and develop clinical decision making based on different prognosis.

Several limitations still existed in our study. Some potential variables and recognized prognostic factors (such as lymphovascular invasion, genetic mutations and etc.) were not available in the SEER database, we failed to incorporate these parameters in this model. Furthermore, the SEER database also lacks information on disease-free survival (DFS). Admittedly, the C-indexes of our nomogram and AJCC staging system was not satisfactory enough (C-index <0.70). That is because our clinical nomogram was based on preoperative data, and did not incorporating other well-known prognostic variables (for example, pTNM categories and clinical management). In addition, certain bias may exist due to the retrospective nature of the study. Further efforts to collect prospective data are warranted to improve the predictive performance and general applicability of our model. By incorporating other prognostic factors in the future, the nomogram model could increase the applicability in clinical practice.

In conclusion, we established and validated a nomogram to predict OS for patients with esophageal adenocarcinoma. The nomogram presented significantly better discrimination than the current AJCC TNM classification. The convenient system provided a reliable tool for predicting individualized survival and have potential implications for treatment strategy making.

## Data Availability Statement

The original contributions presented in the study are included in the article/**Supplementary Material**. Further inquiries can be directed to the corresponding authors.

## Author Contributions

Conception and design: C-yS, YY, H-zS, YS, and JY. (II) Administrative support: JY, Q-fL, and YS. (III) Provision of study materials or patients: X-lL. (IV) Collection and assembly of data: YY and Q-fL. (V) Data analysis and interpretation: C-yS, YY, and Q-fL. (VI) Manuscript writing: All authors. (VII) All authors contributed to the article and approved the submitted version.

## Funding

This study was funded by Natural Science Foundation of Jiangsu Province, Grant/Award Number: BK20181083 and National Natural Science Foundation of China, Grant/Award Number: 81902453.

## Conflict of Interest

The authors declare that the research was conducted in the absence of any commercial or financial relationships that could be construed as a potential conflict of interest.

## Publisher’s Note

All claims expressed in this article are solely those of the authors and do not necessarily represent those of their affiliated organizations, or those of the publisher, the editors and the reviewers. Any product that may be evaluated in this article, or claim that may be made by its manufacturer, is not guaranteed or endorsed by the publisher.
